# Supplementary material for: The interactome of intact mitochondria by cross-linking mass spectrometry provides evidence for coexisting respiratory supercomplexes
Source: Mol Cell Proteomics. 2017 Dec 8;17(2):216–32. doi: 10.1074/mcp.RA117.000470 (PMC5795388; doi:10.1074/mcp.RA117.000470)
Supplement: Supplemental Data [file supp_RA117.000470_133922_0_supp_23978_fzffwf.zip › spectra_annotation/mito_spectra_annotation/417-1-6-1-14-1.pdf]

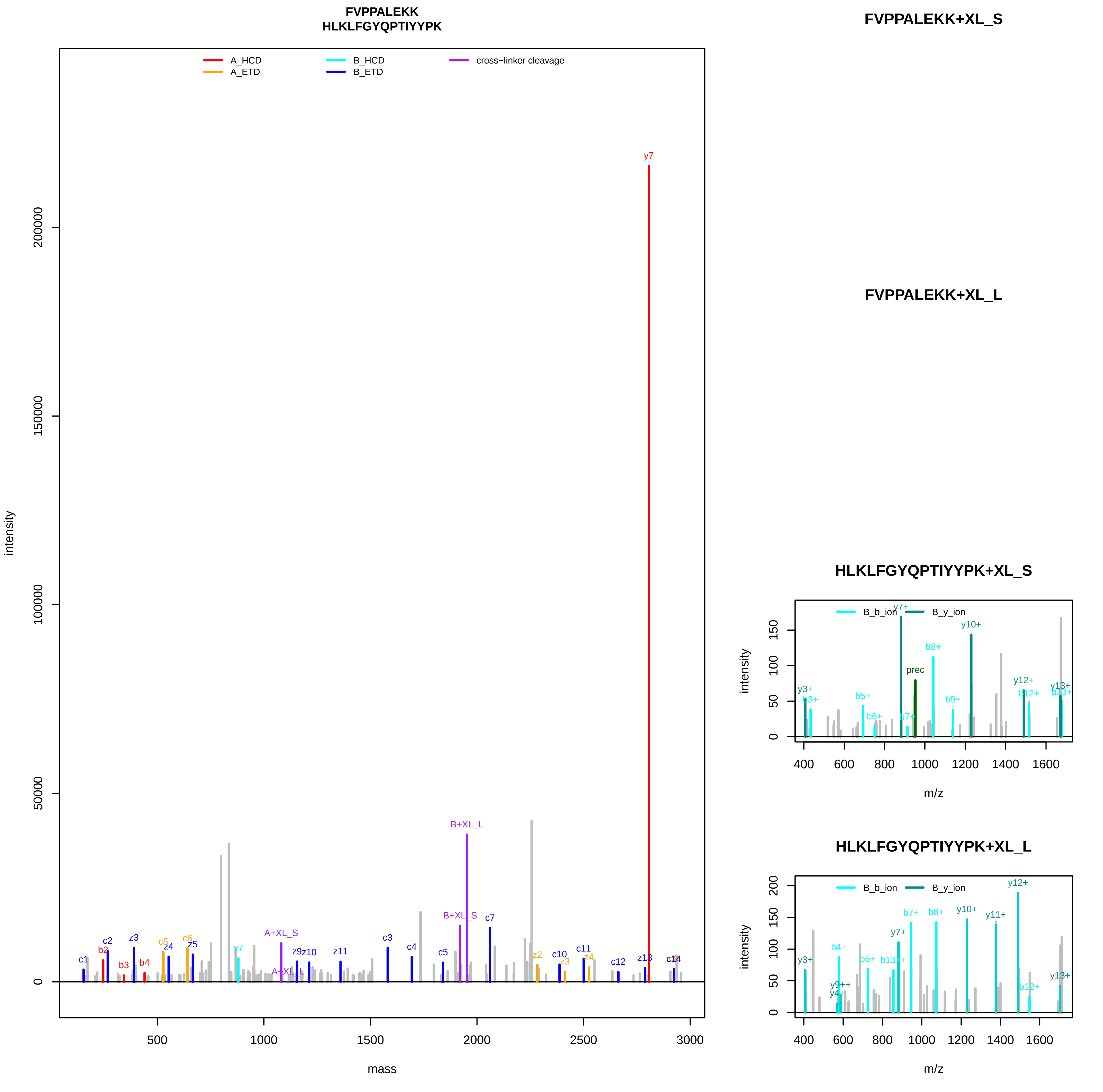
FVPPALEKK
HLKLFGYQPTIYYPK
A_HCD
A_ETD
B_HCD
B_ETD
cross−linker cleavage
intensity
mass
0
50000
100000
150000
200000
500
1000
1500
2000
2500
3000
y7
B+XL_L
B+XL_S
A+XL_S
A+XL_L
c1 b2 c2 b3 z3 b4 c5 z4 c6 z5 y7 z9 z10 z11 c3 c4 c5 c7 z2 z3 c10 c11 z4 c12 z13 c14
FVPPALEKK+XL_S
FVPPALEKK+XL_L
HLKLFGYQPTIYYPK+XL_S
B_b_ion
B_y_ion
y3+ b3+ b5+ b6+ b7+ y7+ prec b8+ b9+ y10+ y12+ b12+ y13+ b13+
m/z
HLKLFGYQPTIYYPK+XL_L
y3+ y4+ y9++ b4+ b5+ b13++ y7+ b7+ b8+ y10+ y11+ y12+ b12+ y13+
400
600
800
1000
1200
1400
1600
